# Assessment of the Relationship between Nurses’ Perception of Ethical Climate and Job Burnout in Intensive Care Units

**DOI:** 10.17533/udea.iee.v38n3e12

**Published:** 2020-11-11

**Authors:** Mozhgan Rivaz, Fatemeh Asadi, Parisa Mansouri

**Affiliations:** 1 Ph.D. in Nursing, Assistant professor. Email: mrivaz@sums.ac.ir mrivaz@sums.ac.ir; 2 M.Sc. Critical Care Nurse .Email: asadi.fa@yahoo.com asadi.fa@yahoo.com; 3 M.Sc. in Nursing. Email: Mansoorip@sums.ac.ir. Corresponding author Mansoorip@sums.ac.ir; 4 School of Nursing and Midwifery, Shiraz University of Medical Sciences, Shiraz, Iran. Shiraz University of Medical Sciences Shiraz Iran

**Keywords:** burnout, professional, intensive care units, nurses, ethics, nursing, agotamiento profesional, unidades de cuidados intensivos enfermeras y enfermeros, ética en enfermería, esgotamento profissional, unidades de terapia intensiva, enfermeiras e enfermeiros, ética em enfermagem

## Abstract

**Objective.:**

To determine the relationship between ethical climate and burnout in nurses working in Intensive Care Units (ICUs).

**Methods.:**

This cross-sectional and multi-center study was conducted among 212 nurses working in adult ICUs of six hospitals affiliated to Shiraz University of Medical Sciences, Iran in 2019. The participants were selected using systematic random sampling technique. Data was collected using valid instruments of Olson's Hospital Ethical Climate Survey (HECS) and Maslach Burnout Inventory (MBI).

**Results.:**

Ethical climate was favorable (3.5±0.6). The intensity (32.2±12.4) and frequency (25.5±12.4) of burnout were high. Ethical climate had significant and inverse relationships with frequency of burnout (r =-0.23, *p*=0.001) and with intensity of burnout (r=-0.186, *p*=0.007). Ethical climate explained 5.9% of burnout. Statistically significant relationships were also found between these factors: age with ethical climate (*p*=0.001), work shifts with burnout (*p*=0.02), and gender and with intensity frequency of burnout in ICU nurses (*p*=0.038). The results of Spearman correlation coefficient showed significant and inverse relationships between ethical climate and job burnout (r=-0.243, *p*<0.001).

**Conclusion.:**

Nurses in ICUs perceived that ethical climate was favorable however, burnout was high. Therefore, burnout can be affected by many factors and it is necessary to support ICU nurses since they undertake difficult and complicated task. It is recommended to assess factors that increase burnout and adopt specific measures and approaches to relieve nursing burnout.

## Resumen

**Objetivo.:**

Determinar la percepción de las enfermeras sobre el clima ético y el agotamiento laboral en las Unidades de Cuidados Intensivos (UCI).

**Métodos.:**

Este estudio de corte transversal y multicéntrico se realizó con 212 enfermeros que trabajaban en UCI para adultos de seis hospitales afiliados a la Universidad de Ciencias Médicas de Shiraz, Irán, en 2019. Los participantes se seleccionaron mediante una técnica de muestreo aleatorio sistemático. Los datos se recolectaron a partir de la utilización de instrumentos Encuesta de Clima Ético en Hospitales (HECS) de Olson y el Inventario de Burnout de Maslach (MBI).

**Resultados.:**

El clima ético fue favorable (3.5±0.6). La intensidad (32.2±12,4) y la frecuencia (25.5±12.4) del agotamiento fueron altas. El clima ético tuvo relaciones significativas e inversas con la frecuencia de Burnout (r=-0.23, *p*=0.001) y con la intensidad del burnout (r=-0.186, *p*=0.007). El clima ético explica el 5.9% del agotamiento. También se encontraron relaciones estadísticamente significativas entre edad y clima ético (*p*=0.001), turnos de trabajo y burnout (*p*=0.02), además de género y frecuencia de intensidad de burnout (*p*=0.038). El coeficiente de correlación de Spearman mostró relaciones significativas e inversas entre el clima ético y el agotamiento laboral (r=-0.243, *p*<0.001).

**Conclusión.:**

Las enfermeras en las UCI percibieron que el clima ético era favorable, sin embargo, el desgaste fue alto. Por tanto, el burnout puede verse influido por muchos factores y es necesario apoyar a estas enfermeras que cumplen una tarea difícil y complicada. Se recomienda evaluar los factores que aumentan el agotamiento y adoptar medidas y enfoques específicos para aliviar el burnout de enfermería.

## Resumo

**Objetivo.:**

Verificar a diferença entre a percepção dos enfermeiros sobre o clima ético e o desgaste no trabalho em Unidade de Terapia Intensiva (UTI).

**Métodos.:**

Este estudo transversal e multicêntrico foi realizado com 212 enfermeiras que trabalham na UTI adulto de seis hospitais afiliados à Universidade de Ciências Médicas de Shiraz, Irão, em 2019. Os participantes foram selecionados por meio de uma técnica de amostragem aleatória sistemática. Os dados foram coletados usando os instrumentos Enquete de Clima Ético em Hospitais (HECS) de Olson e o Inventário Burnout de Maslach (MBI).

**Resultados.:**

O clima ético era favorável (3.5±0.6). A intensidade (32.2±12.4) e a frequência (25.5±12.4) de exaustão foram altas. O clima ético teve relações significativas e inversas com a frequência de Burnout (r=-0.23, *p*=0.001) e com a intensidade de Burnout (r=-0.186, *p*=03007). O clima ético explica 5,9% do esgotamento. Também foram encontradas relações estatisticamente significativas entre: idade e clima ético (*p*=0.001), turnos de trabalho e Burnout (*p*=0,02), além de gênero e frequência da intensidade do Burnout (*p*=0.038). O coeficiente de correlação de Spearman mostrou relações significativas e inversas entre clima ético e Burnout (r=-0243, *p*<0.001).

**Conclusão.:**

As enfermeiras das UTIs perceberam que o clima ético era favorável, porém, o desgaste foi alto. Portanto, o Burnout pode ser influenciado por diversos fatores e é necessário apoiar essas enfermeiras que cumprem uma tarefa difícil e complicada. Recomenda-se avaliar os fatores que aumentam o Burnout e adotar medidas e abordagens específicas para aliviar o Burnout da enfermagem.

## Introduction

Intensive Care Unit (ICU) is one of the most critical and complicated hospital wards where nurses undertake difficult tasks and duties.(1) An increase in the number of ICU patients worldwide poses numerous challenges to the healthcare system including ICU nursing staff retention, heavy workload, and declined health.(2) ICU is characterized by a high level of work -associated stress, which is a factor to increase the risk of burnout.(3) Burnout is a syndrome characterized by emotional exhaustion that results in depersonalization and decreased personal accomplishment at work.(4) Negative physical and psychological consequences of job burnout lead to lower efficiency and reduced staying at work(5) and is strongly associated with increased nurse turnover.(6) Burnout is considered a threat to patient safety because depersonalization may result in poorer interaction with patients. In addition, lack of motivation and impaired cognitive function as a consequence of burnout can hurt patient safety.(4) Nursing practice environment characteristics including resource inadequacy, poor inter-professional collaboration, and lack of supportive management are associated with nurses’ job burnout.(7) The prevalence of burnout is estimated to range from 10%-70% among nurses and 64% of Chinese nurses experienced job burnout.(4) In Iran, the overall prevalence of nursing burnout has been reported to be 25%.(8) 

Favorable workplace climate and interpersonal relationships help to reduce stress and burnout of nurses in an effective manner.(9) Ethical climate refers to the shared perceptions of ethically correct behaviors and way of handling ethically deviated behaviors.(10) Ethical climate is also described as the perception of an atmosphere that increases ethical thoughts, mutual respect and trust in the organization and allows for questioning, discussion, and expression of different views. Positive climate in hospitals may decrease feelings of loneliness and it has a positive impact on productivity and patient satisfaction.(11) Therefore, unfavorable ethical climate might affect how nurses undertake their tasks in ICUs, undermine their performance, alter their behavior and beliefs, and force them to quit their jobs.(10) A positive ethical climate improves the job satisfaction, decreasing turnover and nursing shortages.(12) Therefore, an understanding of the ethical climate, nursing burnout, and nurses’ perception in the workplace help both policy makers and nurse managers to identify and implement effective mechanisms to change, promote, and control the ethical climate. It also paves the way to improve nursing professional performance and affects caring services and nursing profession. Given the importance of these factors and the limited studies in the ICUs, the present study aimed to determine the relationship between ethical climate and burnout in nurses working in ICUs of the hospitals affiliated to Shiraz University of Medical Sciences, Shiraz, Iran.

## Methods

This was a descriptive, cross-sectional, and multi-center study conducted in 2019. The study population consisted of nurses working in 20 adult ICUs of the six teaching hospitals affiliated to Shiraz University of Medical Sciences, Shiraz, Iran from 2018 to 2019. The sample size was calculated as 193 using Cochran formula by taking into account type I error = 0.05, type II error = 0.2 and the correlation between job burnout and ethical climate score found in previous studies.(13) Given the probable sample loss as 10%, 212 questionnaires were distributed among the participants to improve data accuracy. The participants were selected using Systematic Random Sampling technique. A list of nurses working in the studied hospitals was prepared and the participants were selected from the list in a systematic manner. The inclusion criteria were willing to participate in the study, having at least a bachelor’s degree in nursing, and at least one-year work experience in ICU. Exclusion criteria were incomplete questionnaires.

Measures. The data were collected, using a demographic questionnaire, Olson’s Hospital Ethical Climate Survey (HECS), and Maslach Burnout Inventory (MBI). Demographic questionnaire collected data on gender, age, marital status, hospital ward, and work experience in the hospital, work experience in ICUs, type of employment, work shift, and level of education.

Olson’s Hospital Ethical Climate Survey (HECS). The HECS was originally developed in the USA by Olson to measure hospital nurses' perceptions of the ethical climate in their workplace and it was found to have good validation (Cronbach’s alpha: 0.91). The HECS consisted of 26 items in five dimensions of colleagues (4 items), patients (4 items), managers (6 items), physicians (6 items), and hospital (6 items). The items were scored based on a five-point Likert scale ranging from five (Always) to one (Almost never). The total score of the HECS was obtained by calculating the sum of the item scores. The minimum and maximum scores of the HECS range from 26 to 130. Higher scores indicate a positive ethical climate.(14)

An Iranian version of the HECS was used in this study. The Persian version of HECS was translated using forward-backward method and validated by Rivaz et al. (2019). Construct validity of the scale was assessed using exploratory factor analysis. Principal component analysis provided evidence for factorial validity. Internal consistency using Cronbach’s alpha was 0.86 for the total scale and the Cronbach’s alphas for the domains were between 0.63 and 0.92. The stability of the HECS using intra-class correlation coefficient (ICC) was 0.83.(12)

Maslach Burnout Inventory (MBI). MBI was developed by Maslach et al. in 1985 for measuring the burnout in a variety of occupations, including nursing and medical personnel. It consisted of 22 items and 3 dimensions of emotional exhaustion (9 items), depersonalization (5 items), and personal accomplishment (8 items). The items were scored on a 7-point Likert scale ranging from 0 (Never) to 6 (Every day). A total score was calculated for each domain of the MBI. Whereas the scores of emotional exhaustion were ≤17, 18-29 and ≥30 indicated low, average, and high level of burnout. In depersonalization dimension, the scores of ≤5, 6-11 and ≥12 suggest low, average, and severe burnout, respectively. In personal accomplishment dimension, the scores of ≥40, 34-39 and ≤33 reflect low, average, and severe burnout, respectively. According to Maslach and Jackson, the reliability of the MBI range between 0.71 and 0.92.(15) In Iran, several studies have confirmed the validity and reliability of this instrument. Rivaz *et al.* reported Cronbach’s alpha of 0.95 for the whole questionnaire. Construct validity was established using confirmatory factor analysis (CFA). The result confirmed adequate construct validity of the MBI.(7)

Ethical considerations. The study was approved by the Research Ethics Committee of Shiraz University of Medical Sciences (No: IR.SUMS.REC.1397.219). All participants were fully informed about the aim of the study. Written informed consents were obtained from nurses regarding the voluntary nature of their participation. They were also ensured of data confidentiality.

Data Analysis. The data was analyzed using SPSS v. 21. Descriptive analysis statistics were used to describe the variables. Kolmogorov-Smirnov test was used to assess data normality. Bivariate Pearson correlation coefficients (r) were calculated to assess the relationship between ethical climate and job burnout. Mann-Whitney test, Kruskal-Wallis test, and Univariate linear regression were used to assess the relationship between demographic variables, ethical climate, and job burnout. The level of significance was considered <0.05.

## Results

The findings showed that the majority of ICU nurses were females (65.6%), categorized in the 25-35 age group (62.3%), worked in ICUs of internal diseases (38.2%), and had one to ten years of work experience (78.8%). The findings also showed that 70.8% of the nurses had an experience of working in the ICU, and that 52.8% of them were single, and 98.1% of them had bachelor’s degrees in nursing.

The results showed that the total mean score of ethical climate was favorable (3.51± 0.583). Mean scores of physician and hospital dimensions were relatively favorable, and mean scores of manager, colleagues, and patients dimensions were favorable (Table 1).

**Table 1:** Mean scores of ethical climate and its dimensions

Dimensions	Mean±SD
Manager	3.75±0.92
Physician	3.14±0.85
Hospital	3.10±0.84
Colleagues	3.93±0.72
Patients	3.94±0.65
Total score of HECS	3.51±0.58

The results showed that the mean scores of burnout intensity (32.23±12.36) and that of burnout frequency (25.54±12.36) were high and scores of job burnout dimensions including emotional exhaustion, depersonalization, and personal accomplishment were in low level (Table 2).

**Table 2:** Level of burnout intensity and frequency

Dimensions of burnout	Mean±SD	Level	Level
		Category	Frequency (%)
Frequency of emotional exhaustion	21.92±12.06	≤17	95 (45)
		18-29	59 (28)
		≥30	57 (27)
Intensity of emotional exhaustion	24.87±12.85	≤25	114 (54.8)
		26-39	64 (30.8)
		≥40	30 (14.4)
Frequency of depersonalization	7.41±5.38	≤33	137 (65.62)
		34-39	35 (16.7)
		≥40	38 (18.1)
Intensity of depersonalization	9.06±6.97	≤36	120 (57.4)
		37-43	51 (24.4)
		≥43	38 (18.2)
Frequency of personal accomplishment	29.71±9.45	≤5	89 (42.4)
		6-11	75 (35.7)
		≥12	46 (21.9)
Intensity of personal accomplishment	32.56±11.64	≤6	92 (44)
		7-14	76 (36.4)
		≥15	41 (19.6)
Total score of burnout frequency	25.54±12.36		
Total score of burnout intensity	32.23±12.36		

Kolmogorov-Smirnov test results showed that ethical climate, burnout frequency, and burnout intensity variables did not follow normal distribution (*p*-value <0.05).

The results of Spearman correlation coefficient showed significant and inverse relationships between ethical climate and job burnout (r =-0.243, *p*-value = 0.001). In addition, there were significant and inverse relationships between the total score of burnout frequency and the dimensions of ethical climate (manager and hospital). In addition, there were significant and inverse relationships between the total score of burnout intensity and the dimensions of ethical climate (manager, Physician, and Colleagues) (Table 3)

**Table 3:** Relationships between ethical climate and job burnout

Dimensions	Manager	Physician	Hospital	Colleagues	Patients	Ethical climate
Frequency of emotional exhaustion	r = -0.207 *p* = 0.003	r = -0.185 *p* = 0.007	r = -0.125 *p* = 0.071	r = -0.178 *p* = 0.010	r = -0.169 *p* = 0.014	r = -0.238 *p* = 0.001
Intensity of emotional exhaustion	r = -0.133 *p* = 0.056	r = -0.145 *p* = 0.037	r = -0.015 *p* = 0.828	r = -0.135 *p* = 0.052	r = -0.094 *p* = 0.177	r = -0.116 *p* = 0.094
Frequency of depersonalization	r = -0.046 *p* = 0.521	r = -0.045 *p* = 0.721	r = -0.179 *p* = 0.009	r = -0.023 *p* = 0.741	r = -0.052 *p* = 0.454	r = -0.094 *p* = 0.173
Intensity of depersonalization	r = -0.001 *p* = 0.801	r = -0.055 0.428	r = -0.145 *p* = 0.036	r = -0.030 *p* = 0.661	r = -0.004 *p* = 0.859	r = -0.083 *p* = 0.231
Frequency of personal failure	r = -0.208 *p* = 0.003	r = -0.030 *p* = 0.668	r = -0.047 *p* = 0.503	r = -0.122 *p* = 0.078	r = -0.140 *p* = 0.043	r = -0.140 *p* = 0.042
Intensity of personal failure	r = -0.192 *p* = 0.005	-0.089 *p* = 0.202	r = -0.057 *p* = 0.410	r = -0.161 *p* = 0.020	r = -0.132 *p* = 0.058	r = -0.158 *p* = 0.022
Total score of job burnout frequency	r = -0.218 *p* = 0.001	r = -0.127 *p* = 0.065	r = -0.176 *p* = 0.010	r = -0.137 *p* = 0.047	r = -0.110 *p* = 0.111	r = -0.235 *p* = 0.001
Total score of job burnout intensity	r = -0.179 *p* = 0.010	r = -0.155 *p* = 0.025	r = -0.122 *p* = 0.079	r = -0.161 *p* = 0.020	r = -0.093 *p* = 0.180	r = -0.186 *p* = 0.007

Beta regression model showed that ethical climate explained 5.9% of the changes in burnout in nurses. Beta regression coefficient also showed that ethical climate predicted of burnout in nurses. The more favorable the ethical climate, the lower the burnout (Table 4).

**Table 4:** Predicting severity and frequency of job burnout by ethical climate

Variables	R^2^	F-Statistic	F-p-value	B	β	t-Statistic	p-value
Ethical climate and job burnout from nurses’ perspectives	0.059	13.211	0.001	-7.76	-0.243	-3.635	0.001

The results showed a statistically significant relationship between age and ethical climate in nurses (*p*-value = 0.001). The highest mean score of ethical climate was reported in the 25-35 age group. Statistically significant relationships were also found between these factors: work shifts with burnout (*p*=0.02), and gender with intensity frequency of burnout in ICU nurses (*p* =0.038). The highest mean of burnout in nurses was calculated in women with rotating shifts. The relationships between other demographic variables and ethical climate and burnout were not statistically significant (*p*-value>0.05).

## Discussion

The present study aimed to determine the relationship between ethical climate and job burnout in nurses working in ICUs of the hospitals affiliated to Shiraz University of Medical Sciences. The results showed that ethical climate was favorable. Also, the intensity and frequency of burnout were high. Although the ethical climate and the behaviors expected from the nurses were desirable, the level of job burnout was high. It seems that job burnout can be affected by many factors and that it can be a multifactorial variable. Characteristics of ICUs such as caring of critical patients, inadequate safety principles, inappropriate environment **(**artificial lighting, loud noise, and alarm systems ), variation of procedures (suctioning, LOC scoring**,** ICP monitoring, hourly recording), heavy workload, improper nurse-patient ratio, and lack of competent and trained nurses might be involved in job burnout of nurses in ICUs. In line with our study, Barimani *et al.* concluded that ethical climate was favorable.(16) In other studies, nurses also believed that ethical climate in their hospital was above average (11) and moderate. (17). On the contrary, the studies that were carried out in Turkey(18) and the United States(19) reported unfavorable ethical climate and high levels of moral distress. These confounding results might be due to inadequate communication between the ICU teams. The study carried out in the UK showed that spending more time at the patient bedside and less involvement in clinical decisions raise ethical concerns for the nurses.(20)

According to our results, the intensity and frequency of burnout were high. In line with our study, the mean scores of burnout were high in social welfare centers in Greece (21) and in ICU nurses in Spain.(22) However, the level of burnout was moderate in the study on the nurses working in a hospital in Turkey.(23) The results of the present study showed that nurses in 25-35 age group gained the highest ethical climate score. The relationship between sex and work shift and burnout was also significant. The highest burnout score was reported in women and with rotating shifts. This is reasonable since the majority of nurses were categorized in the 25-35 age group and had rotating shifts in this study. The ethical climate score decreased as age of nurses increased in social welfare centers in Greece (21) and in a hospital in the US.(24) Meltzer(24) and Huckabay and Losa *et al.*(25) found out a higher burnout level in nurses with rotating shifts compared to fixed shifts. This is because rotating shifts might decrease patient satisfaction and reduce personal accomplishment of nurses.

The results of the present study showed that burnout decreased as ethical climate became more favorable. A study on ICU nurses with master degrees in Poland showed a decrease in job burnout in favorable ethical climate.(26) However, Elçi *et al.* (2015) found out that ethical climate had no effect on job burnout in financial services workers.(27) Mulki *et al*.(10) and Minamizono *et al.*(28) found out that ethical climate affects the intention to leave, and a positive significant relationship was found between nurse’s hospital climate perception and their moral sensitivity(11) and performance.(29) Unfavorable ethical climate seems to increase job dissatisfaction, stress, burnout, and intentions to leave the job followed by reduced quality of caring services and prolonged hospitalization.

## Conclusion

The findings of the present study showed favorable ethical climate in perception of nurses working in ICUs. Therefore, it is recommended to improve nursing professional performance and patient safety in ICUs by creating and maintaining a favorable ethical climate in the hospital. Despite the favorable ethical climate, burnout was high in this study. Therefore, burnout can be affected by many factors and it is necessary to support ICU nurses since they undertake difficult and complicated tasks. Nursing burnout increases as ethical climate becomes unfavorable followed by declined quality of patient care. Therefore, it is suggested that managers pave the way to make effective plans for the enhancement of ethical climate in the hospital. They should also assess the factors that increase the rate of burnout in ICU nurses and adopt specific measures and approaches to relieve nursing burnout. Furthermore, nursing managers should adopt effective measures to optimize the workplace and enhance professional ethics through job-related and comprehensive life skills training programs to improve nurses’ job satisfaction and quality of patient care.

Limitations of the study. Nurses who complete the questionnaires were tired and impatient after long hour shifts. They were not in desirable physical and mental condition. These factors influenced the accuracy of responses to the questionnaires that could not be controlled by the researcher.
